# Identification of NIFTP-Specific mRNA Markers for Reliable Molecular Diagnosis of Thyroid Tumors

**DOI:** 10.1007/s12022-023-09781-1

**Published:** 2023-09-02

**Authors:** So-Yeon Lee, Jong-Lyul Park, Kwangsoon Kim, Ja Seong Bae, Jae-Yoon Kim, Seon-Young Kim, Chan Kwon Jung

**Affiliations:** 1 Personalized Genomic Medicine Research Center, Korea Research Institute of Bioscience and Biotechnology, 34141 Daejeon, Korea; 2 Graduate School of New Drug Discovery and Development, Chungnam National University, Daejeon, 34134 Korea; 3 Department of Surgery, College of Medicine, The Catholic University of Korea, Seoul, Korea; 4 Department of Hospital Pathology, College of Medicine, The Catholic University of Korea, Seoul, Korea; 5 College of Medicine, Cancer Research Institute, The Catholic University of Korea, Seoul, Korea

**Keywords:** Messenger RNA, NIFTP, Thyroid neoplasms, Thyroid nodule, RNA sequence analysis, RNA biomarker

## Abstract

Non-invasive follicular thyroid neoplasm with papillary-like nuclear features (NIFTP) is a low-risk thyroid tumor with a favorable prognosis. Nonetheless, differentiating NIFTP from other thyroid tumors remains challenging, necessitating reliable diagnostic markers. This study is aimed at discovering NIFTP-specific mRNA markers through RNA sequencing analysis of thyroid tumor tissues. We performed mRNA expression profiling for 74 fresh frozen thyroid tissue samples, including NIFTP and benign and malignant follicular-cell-derived tumors. NIFTP/malignant tumors showed 255 downregulated genes and 737 upregulated genes compared to benign tumors. Venn diagram analysis revealed 19 significantly upregulated and 7 downregulated mRNAs in NIFTP. Akaike information criterion analysis allowed us to select *OCLN*, *ZNF423*, *LYG1*, and *AQP5* mRNA markers. We subsequently developed a predictive model based on logistic regression analysis using these four mRNAs, which we validated in independent samples (*n* = 90) using a qRT-PCR assay. This model demonstrated high accuracy in predicting NIFTP in discovery dataset (AUC (area under the receiver operating characteristic) = 0.960) and the validation dataset (AUC = 0.757). Our results suggest that *OCLN*, *ZNF423*, *LYG1*, and *AQP5* mRNA markers might serve as reliable molecular markers for identifying NIFTP among other thyroid tumors, ultimately aiding in accurate diagnosis and management of NIFTP patients.

**Supplementary Information:**

The online version contains supplementary material available at 10.1007/s12022-023-09781-1.

## Introduction

Follicular cell-derived neoplasms have three categories (i.e., benign tumors, low-risk neoplasms, and malignant neoplasms) according to the fifth edition of the World Health Organization (WHO) classification of endocrine and neuroendocrine tumors [1, 2]. Prior to 2016, non-invasive follicular thyroid neoplasm with papillary-like nuclear features (NIFTP) was classified as a subtype (non-invasive encapsulated follicular variant) of papillary thyroid carcinoma (PTC) [3, 4]. In the current WHO classification, NIFTP is categorized as a low-risk neoplasm, as it poses an extremely low risk of metastatic spread or structural recurrence [1, 2]. Although tumor cells in NIFTP have features that look like PTC, they are not malignant.

Molecular markers can provide additional information to improve the accuracy and reliability of diagnosis, especially in cases where morphological features alone might be insufficient or ambiguous. At the genomic level, NIFTP is classified as a follicular-cell derived thyroid neoplasm with *RAS*-like molecular profiles, whereas PTC is considered a class of *BRAF*-like neoplasms. However, NIFTP shows significant molecular overlaps with other follicular adenomas (FAs), invasive encapsulated follicular variant of PTC (EFVPTC), and follicular thyroid carcinoma (FTC) [1, 2, 5, 6]. These neoplasms exhibit shared molecular profiles, characterized predominantly by *RAS* variants and less frequently by genetic alterations in *EIF1AX*, *EZH1*, *DICER1*, *PTEN*, and *TSHR* genes [1, 7–9]. Additionally, gene fusions involving *PPARG* and *THADA* genes have been identified [1, 6, 8]. In cytologic specimens, NIFTP is often indistinguishable from benign or malignant follicular-cell derived thyroid neoplasms with *RAS*-like molecular profiles [9–12].

Molecular testing, which analyzes mRNA expression patterns, has been utilized to differentiate between benign and malignant tumors in cytology samples. In 2011, a 167-gene classifier utilizing mRNA expression data and machine learning (Afirma Gene Expression Classifier) was introduced for molecular testing of thyroid nodules, to identify benign nodules among indeterminate thyroid nodules detected by cytopathology [13]. A newer version of this gene expression classifier called Afirma Genomic Sequencing Classifier was introduced in 2017 [14]. This classifier employs next-generation sequencing methodology to incorporate 10,196 genes (including 1115 core genes) and machine learning to enable better distinction between benign and malignant nodules [14]. The Genomic Sequencing Classifier was trained to identify NIFTP cases as suspicious [6]. However, there is limited understanding regarding the identification of a set of differentially expressed genes that can distinguish NIFTP from other types of thyroid tumors. Protein biomarkers for diagnosing malignancy in thyroid cytology samples have also been investigated using high-throughput proteomics methods. Advanced computational and bioinformatics tools have been employed to analyze proteomics data, discern differences in protein expression between benign and malignant samples, and reveal potential protein biomarkers [15]. Nevertheless, panels employing proteomics have not been commercialized to date.

The aim of this study was to identify a set of differentially expressed genes that could accurately distinguish NIFTP from other benign and malignant follicular-cell derived thyroid neoplasms.

## Materials and Methods

### Study Subjects

This study received approval from the Institutional Review Board of Seoul St. Mary’s Hospital of the Catholic University of Korea (KC20TISI0766). Samples of thyroid tumor tissue and non-tumor tissue were obtained from the Biobank of Seoul St. Mary’s Hospital. Demographic and baseline characteristics of patients in the discovery and validation datasets are summarized in Table 1.

**Table 1:** Baseline characteristics of patients in the discovery and validation populations

**Characteristics**	**Discovery dataset**	**Validation dataset**
Sample no.	74	90
Age years at diagnosis, mean (range)	48 (19–78)	50 (19–80)
Sex		
Female	50	73
Male	24	17
Tumor size (cm), mean (range)	3.0 (1.4–6.0)	3.2 (1.2–12.0)
Diagnosis		
Normal thyroid tissue	0	12
Follicular nodular disease	10	13
Follicular adenoma	24	18
NIFTP	14	10
IEFVPTC, minimally invasive	0	14
PTC, classic	6	6
PTC, encapsulated classic	4	3
PTC, tall cell	0	3
PTC, diffuse sclerosing	1	1
PTC, encapsulated solid/trabecular	1	0
PTC with *BRAF* V600E	0	11
PTC with *BRAF* wild-type	12	2
FTC, minimally invasive	11	7
FTC, encapsulated angioinvasive	3	2
FTC, widely invasive	0	1

*NIFTP* non-invasive follicular thyroid neoplasm with papillary-like nuclear features, *IEFVPTC* invasive encapsulated follicular variant of papillary thyroid carcinoma, *PTC* papillary thyroid carcinoma, *FTC* follicular thyroid carcinoma

For the discovery dataset, we collected 74 fresh frozen tissue samples to profile mRNA expression levels. These samples included 10 cases of thyroid follicular nodular disease (FND), 24 cases of follicular adenoma (FA), 14 cases of non-invasive follicular thyroid neoplasm with papillary-like nuclear features (NIFTP), 6 cases of classic papillary thyroid carcinoma (PTC), 6 cases of other PTC subtypes (3 encapsulated classic PTCs with predominant follicular growth, 2 invasive encapsulated solid/trabecular PTCs, 1 diffuse sclerosing PTC), and 14 cases of follicular thyroid carcinoma (FTC).

The validation dataset comprised a total of 90 fresh frozen thyroid samples, including normal thyroid tissue (*n* = 12), FND (*n* = 13), FA (*n* = 18), NIFTP (*n* = 10), FTC (*n* = 10), invasive encapsulated follicular variant of papillary thyroid carcinoma (IEFVPTC, *n* = 14), and PTC (*n* = 13). The specific histologic subtypes are described in Table 1. These tissue samples were obtained from different years compared to the samples in the discovery dataset.

All tumor samples utilized in this study were evaluated for the status of the *BRAF* V600E variant using real-time PCR PNA clamping technology (Panagene, Daejeon, Korea) [16]. All samples in the discovery dataset were deliberately selected to be negative for the *BRAF* V600E variant. This intentional inclusion of *BRAF* V600E negative tumors in the discovery dataset aimed to exclude any gene expression changes induced by the *BRAF* V600E. This distinction is particularly relevant since NIFTP and other follicular-patterned tumors (FND, FA, and FTC) do not possess the *BRAF* V600E mutation. In contrast, the validation dataset included 11 PTCs with the *BRAF* V600E variant. This decision was made because the selected mRNA markers needed to be effective in differentiating NIFTP from other follicular cell-derived neoplasms, including PTCs with *BRAF* V600E.

### Total RNA Preparation and mRNA Sequencing

Total RNAs were isolated from fresh-frozen tissues using a RNeasy Kit (Qiagen, Carlsbad, CA, USA) following the manufacturer’s instructions. The quantity and quality of obtained total RNAs were evaluated using an ND-1000 spectrophotometer (Thermo Fisher Scientific, Waltham, MA, USA). RNA integrity number (RIN) was estimated using a 2100 Agilent Bioanalyzer (Agilent Technologies, Waldbronn, Germany). Approximately 1 μg of total RNAs was utilized for library preparation with an Illumina TruSeq Stranded Total RNA Library Prep Kit (San Diego, CA, USA). An Illumina NovaSeq 6000 System sequencing instrument was employed to perform 101-bp paired-end sequencing, generating following the manufacturer’s instructions.

### mRNA Sequencing Data Analysis in the Discovery Dataset

We eliminated TruSeq small RNA adapters from sequenced reads using Trimmomatic software (v. 0.38). Remaining sequence data were mapped to the human genome (GRCh38) for quantification using STAR software (v. 2.7.a) [17]. To identify differentially expressed mRNA, the edgeR software was employed [18]. We utilized the default parameter configuration for all programs. The mRNA-seq dataset is available from Korean Nucleotide Archive (KoNA, https://kobic.re.kr/kona) and Sequence Read Archive (SRA, https://www.ncbi.nlm.nih.gov/sra) public databases (accession number: PRJKA220514 and PRJNA918826, respectively).

### mRNA Expression Levels by qRT-PCR in the Validation Dataset

In the validation dataset, mRNA expression levels were evaluated using quantitative reverse-transcription PCR (qRT-PCR). To assess the expression levels of selected candidate mRNAs for the identification of NIFTP, 2 µg of total RNA from each specimen was converted into cDNA using the iScript reverse transcriptase kit (Bio-Rad, Hercules, CA). The subsequent real-time RT-PCR procedure was performed using the iQ SYBR Green Supermix (Bio-Rad) on a CFX96 real-time PCR system (Bio-Rad). The quantified mRNA levels were normalized to β-actin levels. Detailed information about primer sequences and the experimental conditions used are provided in Table S1.

### Public mRNA Sequencing Data Analysis

We obtained public mRNA-sequencing data of thyroid samples from The Cancer Genome Atlas (TCGA) dataset (https://portal.gdc.cancer.gov/) to confirm mRNA expression patterns of candidate mRNA markers.

### Statistical Analysis

To evaluate significance of difference in gene expression between benign and NIFTP/malignant tumor tissues, Student’s *t*-test or analysis of variance (ANOVA) was used. We performed hierarchical clustering using Multiple Experiment Viewer (MEV) software (version 4.8.1) and Pearson’s correlation method [19]. For Kyoto Encyclopedia of Genes and Genomes (KEGG) Gene Ontology (GO) enrichment analysis, we used the ShinyGO tool [20]. Using ROCR package of R software (version 4.2.2), we calculated receiver operating characteristic (ROC) and area under the ROC curve (AUC) for each mRNA marker. We performed logistic regression for the combination of mRNA markers to identify NIFTP from other thyroid tumors. The prediction score was calculated by multiplying expression level of each mRNA with its corresponding regression coefficient and then summing them up in a linear combination. We estimated optimal cutoff values maximizing sensitivity and specificity between low and high levels of mRNA expression using ROC curve analysis. Results with *p* values < 0.05 were considered significant.

## Results

### Global Differential Gene Expression Between Benign and NIFTP/Malignant Thyroid Tumors

**Fig. 1:**
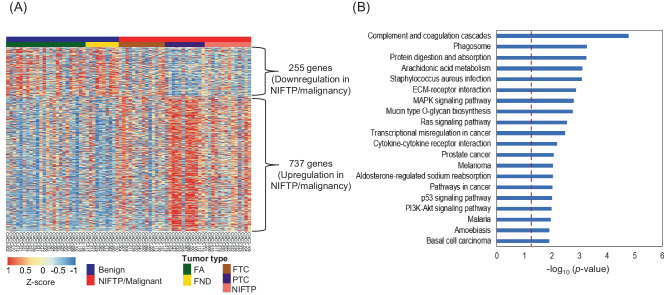
mRNA expression profiling for fresh frozen thyroid tumor tissues (*n* = 74). **A** Unsupervised hierarchical cluster analysis was conducted to examine the expression levels of 255 downregulated and 737 upregulated genes in NIFTP and malignancy, which were identified using the edgeR software. RNA expression levels were normalized by Z-score. **B** KEGG pathway enrichment analysis was performed for differentially expressed genes (DEGs) between benign and NIFTP/malignant thyroid tumors

In this study, we conducted mRNA expression profiling of 74 fresh frozen thyroid tissues, including thyroid benign (FND and FA), NIFTP, and malignant (PTC and FTC) tumors. Notably, we only included PTCs that lacked *BRAF* V600E variant. To identify differentially expressed genes (DEGs), we applied two criteria: (1) *p* value < 0.05 and (2) log_2_ fold change > 0.5 between benign thyroid tumors (FA and FND) and NIFTP/malignancy (FTC and PTC). Based on these criteria, we identified 255 downregulated and 737 upregulated genes in NIFTP/malignancy compared to benign tumors. Figure 1A shows results of unsupervised hierarchical clustering using these DEG candidates.

Next, we performed KEGG pathway enrichment analysis using DEGs between benign tumors and NIFTP/malignancy and found several cancer-associated pathways, including ECM-receptor interaction, MAPK signaling pathway, Ras signaling pathway, pathway in cancer, and p53 signaling pathway (Fig. 1B). Our results demonstrate that RNA-seq platform can successfully identify DEGs between benign and NIFTP/malignant thyroid tumors.

### Identification of Differentially Expressed mRNAs in NIFTP

**Fig. 2:**
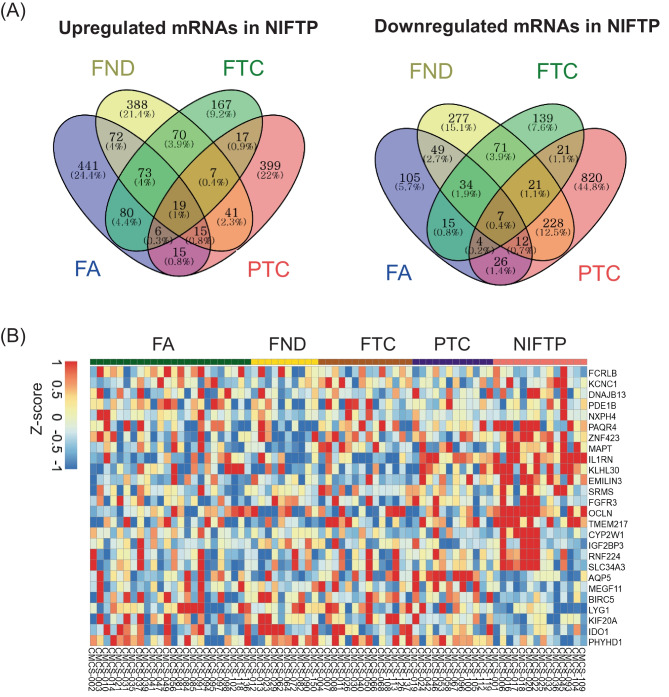
Identification of NIFTP-specific mRNA markers in the discovery dataset. **A** Comparative analysis of differentially expressed mRNAs between NIFTP and other subtypes (FND, FA, FTC, and PTC) revealed 19 upregulated and 7 downregulated mRNAs in NIFTP. **B** A heatmap of differentially expressed mRNAs was generated for the thyroid tumor subgroup. RNA expression levels were normalized to *Z*-score

We analyzed differentially expressed mRNAs between various tumor subgroups (FND vs. NIFTP, FA vs. NIFTP, FTC vs. NIFTP, and PTC vs. NIFTP) to identify mRNA markers exclusive to NIFTP. Using Venn diagrams, we identified 19 significantly upregulated mRNAs and 7 significantly downregulated mRNAs in NIFTP (Fig. 2A). We performed unsupervised hierarchical clustering of candidate mRNA markers. Results are shown in Fig. 2B.

To further refine our list of NIFTP-specific candidate mRNA markers, we analyzed the TCGA dataset. The TCGA project did not enroll cases of NIFTP since the tumor was not yet recognized as a distinct entity until after the TCGA project was completed in 2016 [3, 21]. In this study, we reviewed whole slide images available in the TCGA dataset and observed that encapsulated follicular subtype of PTC might encompass cases that could potentially be classified as NIFTPs. We excluded follicular PTCs with *BRAF* V600E-like molecular alterations. Although some of the encapsulated follicular PTCs might not meet the criteria for NIFTP diagnosis, they were nevertheless instrumental in validating our results. Out of the 26 candidate mRNAs identified in our own samples, 12 of them exhibited distinct expression levels between the follicular subtype and other subtypes of PTC in the TCGA dataset.

Out of the 12 selected mRNAs, 8 (*KCNC1*, *PDE1B*, *NXPH4*, *ZNF423*, *MAPT*, *SRMS*, *OCLN*, and *IGF2BP3*) were upregulated and 4 (*AQP5*, *LYG1*, *IDO1*, and *PHYHD1*) were downregulated in NIFTP as shown in Table 2. These 12 mRNAs were incorporated into subsequent downstream analyses.

**Table 2:** Top 12 candidate mRNA markers to discriminate NIFTP from other thyroid tumors in the discovery dataset

mRNAs	Expression in NIFTP	NIFTP vs. PTC			NIFTP vs. FTC			NIFTP vs. FA			NIFTP vs.FND		
		*P*	Fold change (log2 scale)	Average CPM (log2 scale)	*P*	Fold change (log2 scale)	Average CPM (log2 scale)	*P*	Fold change (log2 scale)	Average CPM (log2 scale)	*P*	Fold change (log2 scale)	Average CPM (log2 scale)
*KCNC1*	UP	2.94 × 10^–3^	2.394	0.779	1.35 × 10^–2^	1.665	0.898	3.31 × 10^–4^	1.985	0.763	3.31 × 10^–4^	2.406	0.811
*PDE1B*	UP	1.35 × 10^–2^	1.981	3.611	2.74 × 10^–2^	1.705	3.579	6.47 × 10^–3^	1.622	3.612	6.47 × 10^–3^	2.326	3.458
*NXPH4*	UP	4.29 × 10^–4^	6.118	0.855	8.78 × 10^–4^	5.249	0.874	4.56 × 10^–5^	4.588	0.96	4.56 × 10^–5^	5.968	0.883
*ZNF423*	UP	9.43 × 10^–3^	0.836	3.256	5.84 × 10^–3^	0.844	3.225	2.51 × 10^–3^	0.882	3.057	2.51 × 10^–3^	1.008	3.148
*MAPT*	UP	5.68 × 10^–3^	1.917	2.863	9.83 × 10^–3^	1.478	3.09	9.61 × 10^–5^	1.912	2.731	9.61 × 10^–5^	2.809	2.785
*SRMS*	UP	8.67 × 10^–4^	3.768	2.324	1.48 × 10^–2^	3.430	2.388	2.48 × 10^–6^	3.720	2.135	2.48 × 10^–6^	3.81	2.375
*OCLN*	UP	2.01 × 10^–3^	0.710	5.537	1.05 × 10^–2^	0.647	5.503	7.86 × 10^–4^	0.565	5.504	7.86 × 10^–4^	0.609	5.550
*IGF2BP3*	UP	3.63 × 10^–4^	4.505	2.68	5.00 × 10^–4^	3.918	2.75	8.86 × 10^–3^	2.418	2.62	8.86 × 10^–3^	3.625	3.071
*AQP5*	DOWN	3.94 × 10^–6^	−3.987	3.38	2.29 × 10^–4^	−3.603	2.838	6.99 × 10^–4^	−3.356	2.779	6.99 × 10^–4^	−3.593	2.175
*LYG1*	DOWN	2.47 × 10^–2^	−0.575	1.429	1.48 × 10^–2^	−0.699	1.472	6.87 × 10^–3^	−0.612	1.494	6.87 × 10^–3^	−0.506	1.424
*IDO1*	DOWN	1.44 × 10^–2^	−2.125	1.27	4.09 × 10^–2^	−1.549	1.326	3.41 × 10^–3^	−3.029	1.491	3.41 × 10^–3^	−3.136	1.670
*PHYHD1*	DOWN	5.55 × 10^–3^	−0.886	5.224	6.82 × 10^–3^	−0.996	5.229	3.28 × 10^–2^	−0.758	5.144	3.28 × 10^–2^	−0.929	5.148

*NIFTP* non-invasive follicular thyroid neoplasm with papillary-like nuclear features, *PTC* papillary thyroid carcinoma, *FTC* follicular thyroid carcinoma, *FA *follicular adenoma, *FND* thyroid follicular nodular disease

### Combination of mRNA Markers to Identify NIFTP

**Fig. 3:**
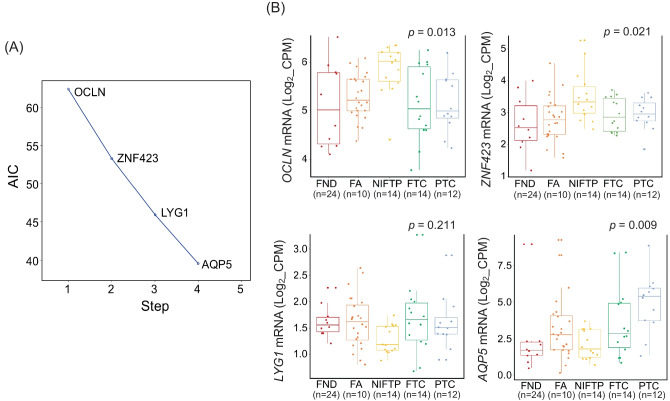
Multiple mRNA marker selection for identification of non-invasive follicular thyroid neoplasm with papillary-like nuclear features (NIFTP) in the discovery dataset. **A** Akaike information criterion (AIC) analysis was performed using 12 candidate mRNA markers to discriminate NIFTP with Blorr R software packages. Genes selected by the AIC included *OCLN*, *ZNF423*, *LYG1*, and *AQP*5. **B** Expression patterns of the 12 selected four mRNA markers in each thyroid tumor were analyzed using RNA expression levels normalized to log_2_ CPM. ANOVA analysis was used to estimate statistical significance

To determine the optimal combination of mRNA markers for identifying NIFTP, we conducted an Akaike information criterion (AIC) analysis using the top 12 candidate mRNA markers. We ultimately selected *OCLN* (occludin), *ZNF423* (zinc finger protein 423), *LYG1* (lysozyme g1), and *AQP5* (aquaporin 5) mRNA markers based on their expression pattern in the discovery dataset (Fig. 3).

To estimate the accuracy of these four NIFTP-specific mRNA markers, we performed ROC analysis and calculated AUC values. AUC values for *OCLN*, *ZNF423*, *LYG1*, and *AQP5* were 0.80, 0.73, 0.73, and 0.68, respectively (Table 3).

**Table 3:** Diagnostic performance of selected mRNA markers to discriminate non-invasive follicular thyroid neoplasm with papillary-like nuclear features from other thyroid tumors in the discovery dataset

mRNA	Cut-off value	Sensitivity	Specificity	PPV	NPV	AUC
*OCLN*	5.436	92.9	61.7	36.1	97.4	0.802
*ZNF423*	2.718	92.9	45	28.3	96.4	0.733
*LYG1*	1.568	92.9	51.7	31.0	96.9	0.727
*AQP5*	3.689	100	38.3	27.5	100	0.681
Combination of the four mRNA markers	23.872	100	88.3	66.7	100	0.960

*PPV* positive predictive value, *NPV* negative predictive value, *AUC* area under the receiver operating characteristic curve

**Fig. 4:**
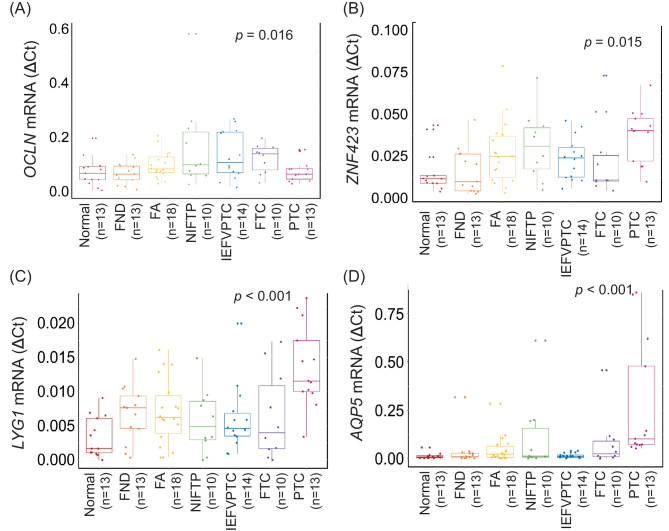
Receiver operating characteristic (ROC) analyses of a multiple logistic regression model to discriminate non-invasive follicular thyroid neoplasm with papillary-like nuclear features (NIFTP) from other types of thyroid tumors in discovery dataset (**A**) and validation dataset (**B**)

We then developed the following equation for predicting NIFTP using a logistic regression analysis of the four mRNAs: prediction score = −25.120 + (3.797 × expression level of *OCLN*) + (3.690 × expression level of *ZNF423*) + (−5.229 × expression level of *LYG1*) + (−0.756 × expression level of *AQP5*). The prediction model had an AUC value of 0.960 in the discovery dataset (Fig. 4A).

### Validation of mRNA Markers to Identify NIFTP in an Independent Cohort

**Fig. 5:**
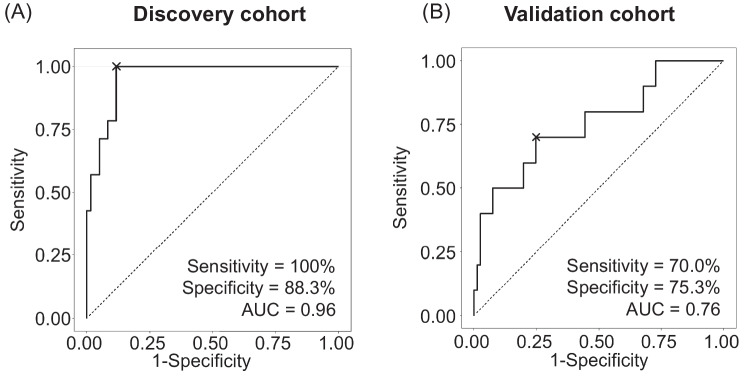
Expression levels of selected four mRNA markers in validation dataset using qRT-PCR. Expression levels of *OCLN* (**A**), *ZNF423* (**B**), *LYG1* (**C**), and *AQP5* (**D**) are shown in relation to normal thyroid tissue and various tumor types. FND, thyroid follicular nodular disease; FA, follicular adenoma; NIFTP, non-invasive follicular thyroid neoplasm with papillary-like nuclear features; IEFVPTC, invasive encapsulated follicular variant of papillary thyroid carcinoma; FTC, follicular thyroid carcinoma; PTC, papillary thyroid carcinoma

The four NIFTP-specific mRNA markers were validated in independent samples using a qRT-PCR assay. These samples encompassed normal thyroid tissue, FND, FA, NIFTP, FTC, IEFVPTC, and PTC. All four mRNA markers demonstrated significant differential expression across these tumor types (Fig. 5). We then generated a prediction equation for NIFTP using a logistic regression analysis of these four mRNAs to assess their capacity to distinguish NIFTP from other tumor types: prediction score = −3.722 + (9.950 × expression level of *OCLN*) + (70.667 × expression level of *ZNF423*) - (273.325 × expression level of *LYG1*) + (2.752 × expression level of *AQP5*). The prediction model demonstrated a promising AUC value in the validation dataset (AUC = 0.757, Fig. 4B). The performance of the model is summarized in Table 4.

**Table 4:** Diagnostic performance of selected mRNA markers to discriminate non-invasive follicular thyroid neoplasm with papillary-like nuclear features from other thyroid tumors in the validation dataset

mRNA	Cut-off value	Sensitivity	Specificity	PPV	NPV	AUC
*OCLN*	0.177	40	88.9	30.8	92.3	0.626
*ZNF423*	0.037	50	72.8	18.5	92.2	0.598
*LYG1*	0.003	50	71.6	17.9	92.1	0.575
*AQP5*	0.193	30	91.4	30.0	91.4	0.510
Combination of the four mRNA markers	1.374	70	75.3	25.9	95.3	0.757

*PPV* positive predictive value, *NPV* negative predictive value, *AUC* area under the receiver operating characteristic curve

Our findings suggest that these four mRNA markers can serve as reliable molecular markers for identifying NIFTP among other thyroid tumors.

## Discussion

We identified 19 significantly upregulated mRNAs and 7 significantly downregulated mRNAs in NIFTP compared to various other tumor subgroups in the discovery cohort. Further analyses using TCGA dataset and AIC method led us to select *OCLN*, *ZNF423*, *LYG1*, and *AQP5* mRNA markers as optimal markers for identifying NIFTP. In addition, ROC analysis showed good accuracy for predicting NIFTP using these four mRNA markers in discovery and validation datasets. Our results suggest that these four mRNA markers can serve as reliable molecular markers for identifying NIFTP among other thyroid tumors.

Developing mRNA markers for NIFTP could potentially help differentiate it from other follicular cell-derived thyroid tumors with overlapping morphological features, which can have different clinical management and prognostic implications. These mRNA markers might help reduce overtreatment of indolent tumors and guide more appropriate therapeutic interventions for patients with thyroid cancer. Identification of specific mRNA markers might provide insights into the underlying biology of NIFTP and help further our understanding of its pathogenesis. Additionally, investigating functional roles of these candidate markers in thyroid tumor development and progression could provide insights into molecular mechanisms underlying NIFTP and might reveal potential therapeutic targets for treating thyroid tumors.

In NIFTP, of the four mRNAs investigated, two (*OCLN* and *ZNF423)* exhibited upregulation while the other two (*LYG1* and *AQP5)* displayed downregulation. To date, no studies have examined the roles of these genes in the development or progression of thyroid tumors. Nonetheless, in other cancer types, these genes have been implicated in functioning as either oncogenes or tumor suppressors, depending on the specific tumor context. *OCLN* encodes a protein called occludin, which is an integral membrane protein and a key component of tight junctions between cells [22]. Tight junctions are essential for maintaining cell polarity and regulating the passage of molecules between cells. Downregulation of *OCLN* has been observed in clear cell renal cell carcinoma [23], hepatocellular carcinoma [24], breast cancer [25], endometrial cancer, and lung cancer [26]. However, *OCLN* is overexpressed in bladder cancer. It regulates angiogenesis in bladder cancer [27]. *ZNF423* is a transcription factor that plays a role in regulating the expression of various target genes involved in development, cell differentiation, and DNA damage response [28]. *ZNF423* has been implicated in the regulation of tumor growth, cell proliferation, and apoptosis [29]. Altered *ZNF423* expression has been reported in different cancers, including breast cancer [30], ovary cancer [30], and neuroblastoma [31]. Its role in cancer progression might be context-dependent, acting as either an oncogene or tumor suppressor depending on the specific tumor type and cellular context [32]. *LYG1* belongs to lysozyme G family. Its function in mammalian cells and its role in cancer are not well understood yet. However, *LYG1* may play a role in antitumor function by promoting the activation, proliferation, and function of CD4 + T cells in tumor microenvironment [33]. *AQP5* encodes a water channel protein that is responsible for facilitating water transport across cell membranes. Aquaporins, including AQP5, have been implicated in various aspects of cancer biology, such as cell migration, proliferation, and angiogenesis [34–36]. Altered *AQP5* expression has been observed in multiple cancer types, including lung cancer [34], breast cancer [35], and colorectal cancer [36]. Overexpression of *AQP5* has been associated with increased tumor growth, metastasis, and poor prognosis in some cancer types [37], while its downregulation has been linked to reduced tumor growth and metastasis in non-small cell lung cancer cells [38].

This study is aimed at identifying NIFTP-specific candidate mRNA markers by analyzing both our own datasets and the publicly available TCGA dataset. While the TCGA project did not enroll cases of NIFTP due to its recognition as a distinct entity after the project’s completion, we were able to leverage the TCGA dataset by reviewing whole slide images and selecting encapsulated follicular subtype of PTCs that could potentially be classified as NIFTPs. This approach allowed us to refine our list of candidate mRNA markers. It is important to acknowledge that some of the identified cases in the TCGA dataset might not meet the strict criteria for NIFTP diagnosis. Given that this limitation could potentially affect the specificity of our findings, we primarily utilized the TCGA dataset to narrow down the list of mRNA markers. The diagnostic performance of the final four selected markers was then validated within an independent cohort from our study. This supports the validity of our results and highlights the potential of these markers for differentiating NIFTP from other thyroid tumors.

In this study, we observed the remarkable diagnostic performance of four mRNA markers within the discovery dataset. However, when these findings were subjected to validation in an independent cohort, there was a decrease in diagnostic performance. Several factors could contribute to this disparity in results. First, it is common to observe optimized results within the discovery dataset because the markers were derived and tested within the same dataset. However, the validation dataset introduces real-world scenarios, which could lead to biological variability and possibly different pre-analytical and analytical conditions, thereby influencing the markers’ performance. Second, there were differences in the methodologies employed for analyzing mRNA expression levels between datasets. Specifically, next-generation sequencing was used in the discovery dataset, while qRT-PCR was employed for the validation dataset. Third, it is plausible that the selected mRNA markers are not as universally applicable as initially anticipated. While they demonstrated impressive performance under the controlled conditions of the discovery dataset, their applicability might have been overestimated when considering a broader, more diverse population, as represented in the validation dataset. This suggests that there may be a need to identify additional markers to enhance diagnostic accuracy across varied cohorts. The decreased performance in the validation dataset does not invalidate our findings but suggests that additional refinement and validation of the markers are necessary. Future studies should focus on further improving the diagnostic accuracy of these markers, combining them into a multi-marker panel, or integrating them with clinicopathological parameters in a diagnostic algorithm. Despite these limitations, our study represents an important step forward in the search for reliable, non-invasive diagnostic markers for NIFTP. Our findings provide a foundation upon which further research can build, bringing us closer to the goal of a more accurate diagnosis of low-risk thyroid neoplasms.

## Conclusion

Our study provides evidence that *OCLN*, *ZNF423*, *LYG1*, and *AQP5* mRNA markers can serve as reliable molecular markers for identifying NIFTP among other thyroid tumors. Further research is needed to validate these findings in larger patient cohorts and explore molecular mechanisms underlying the roles of these markers in NIFTP development. Ultimately, implementation of these markers in clinical practice could improve diagnostic accuracy and inform more tailored treatment strategies for patients with thyroid tumors.

### Supplementary Information

Below is the link to the electronic supplementary material.

Supplementary file1 (DOCX 16 KB)

## Data Availability

The mRNA-seq dataset is available in the Korean Nucleotide Archive (KoNA, https://kobic.re.kr/kona) and Sequence Read Archive (SRA, https://www.ncbi.nlm.nih.gov/sra) public databases under accession numbers PRJKA220514 and PRJNA918826.
