# Supplementary material for: Identification of NIFTP-Specific mRNA Markers for Reliable Molecular Diagnosis of Thyroid Tumors
Source: Endocr Pathol. 2023 Sep 2;34(3):311–22. doi: 10.1007/s12022-023-09781-1 (PMC10511606; doi:10.1007/s12022-023-09781-1)
Supplement: Supplementary file 1 — Supplementary file1 (DOCX 16 KB) [file 12022_2023_9781_MOESM1_ESM.docx]

Table S1. Primer sequences used for real-time quantitative reverse transcriptase PCR (qRT-PCR) of selected mRNA genes

| Primer name | Direction | Sequence | Annealing temp./  number of cycles |
| --- | --- | --- | --- |
| β-Actin | Forward | 5’-CAAGAGATGGCCACGGCTGCT-3’ | 60℃ / 40 |
|  | Reverse | 5’-TCCTTCTGCATCCTGTCGGCA-3’ | 60℃ / 40 |
| OCLN | Forward | 5’-GCTACTGGACTCTACGTGGA-3’ | 60℃ / 40 |
|  | Reverse | 5’-GGGGCTGCTCATCATAAATGT-3’ | 60℃ / 40 |
| ZNF423 | Forward | 5’-GTTTGAGAGTGCCGAAGACC-3’ | 60℃ / 40 |
|  | Reverse | 5’-TCGAAGGTCATCTGGCACTT-3’ | 60℃ / 40 |
| LYG1 | Forward | 5’-TGTGGGATTGGAAGACGTCA-3’ | 60℃ / 40 |
|  | Reverse | 5’-GCTAGTCCTATCGCCCATGT-3’ | 60℃ / 40 |
| AQP5 | Forward | 5’-GGTGGAGCTGATTCTGACCT-3’ | 60℃ / 40 |
|  | Reverse | 5’-CGATTCATGACCACCGCAG-3’ | 60℃ / 40 |
